# Heterotopic Endometriosis in the Inguinal Region: A Case Report and Literature Review

**Published:** 2019-12-03

**Authors:** Tae Nagama, Natsuko Kakudo, Michika Fukui, Takashi Yamauchi, Toshihito Mitsui, Kenji Kusumoto

**Affiliations:** Department of Plastic and Reconstructive Surgery, Kansai Medical University, Osaka, Japan

**Keywords:** heterotopic endometriosis, inguinal region, subcutaneous mass, pain, magnetic resonance imaging

## DESCRIPTION

A 41-year-old woman presented with a slow-growing subcutaneous tumor for 10 years in the right inguinal region. Her previous surgical history was right inguinal hernia 14 years ago.

**Figure 1:**
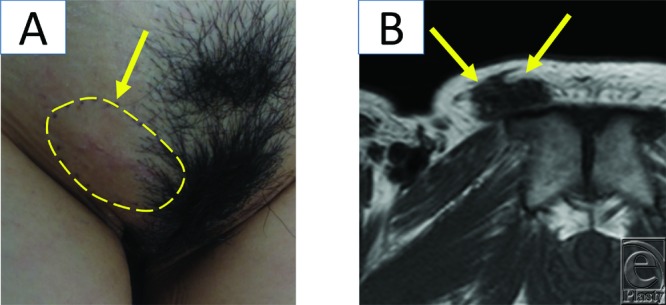
(*a*) Preoperative findings. A 41-year-old woman with an elastic, hard, and immobile 4 × 3.5-mm mass on the right part of the mons pubis. She had superficial pain on and around the tumor site. (*b*) Signal intensity of the tumor in the right inguinal region on magnetic resonance images. In this T1-weighted image, the tumor shows low signal intensity.

A physical examination revealed an elastic, hard, and immobile 4 × 3.5-cm tumor. Superficial pain developed on and around the tumor site (Fig 1*a*). Magnetic resonance imaging (MRI) showed low signal intensity on T1- (Fig 1*b*) and T2-weighted images.

**Figure 2:**
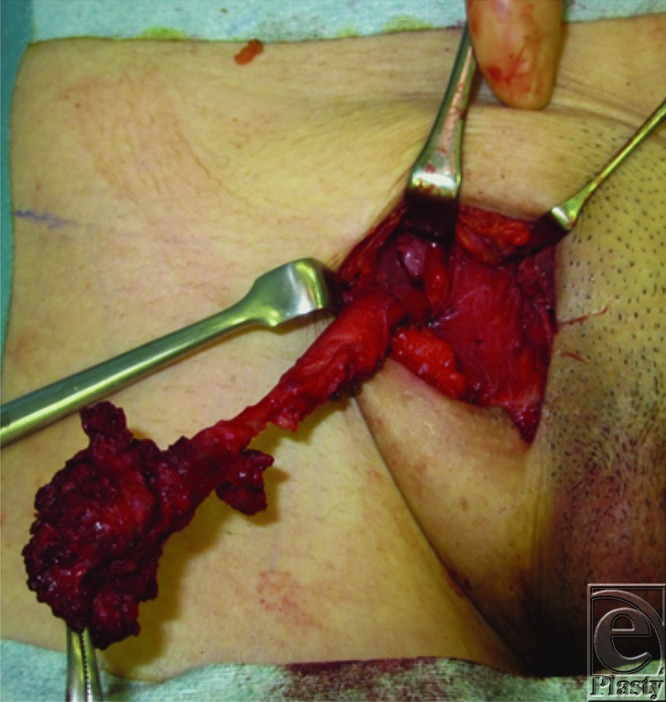
Operative findings. We carefully dissected the tumor and excised between the tumor and transition part of the round ligament of the uterus.

**Figure 3:**
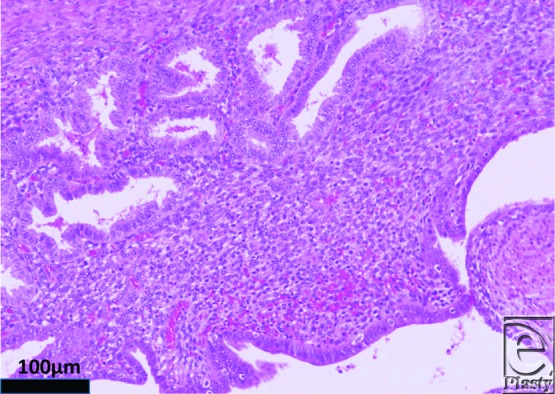
Histological findings of the excised tumor. Hematoxylin-eosin staining shows hyperplasia of the endometrial glands, associated with stroma cells (original magnification ×100).

During surgery, the tumor was pedunculated in the medial direction, penetrated the external oblique fascia, and adhered to the round ligament of the uterus. The external oblique fascia was removed. The tumor was carefully dissected and excised between the tumor and transition part of the round ligament (Fig 2). A histopathological examination with hematoxylin-eosin staining confirmed hyperplasia of the endometrial glands, associated with stroma cells (Fig 3), which was diagnosed as endometriosis.

There has been no pain or tumor recurrence 5 years after surgery.

## QUESTIONS

1. How often does heterotopic endometriosis occur in the inguinal region?
2. What are the clinical symptoms of heterotopic endometriosis?
3. Are there any useful approaches to diagnose heterotopic endometriosis?
4. What is the differential diagnosis of heterotopic endometriosis?

## DISCUSSION

Heterotopic endometriosis is a benign disorder that is defined as the presence of the endometrium or endometrial-like tissue outside the myometrium.^1^

The prevalence of heterotopic endometriosis is 1.2% to 1.5%, with a peak being observed between 35 and 44 years of age.^2^ Heterotopic endometriosis commonly occurs in the pelvic cavity. Unusual sites of endometriosis are the bladder, intestines, surgical scars, diaphragm,^3^ and groin.^4^^,^^5^ The incidence rate of heterotopic endometriosis in the inguinal region is 0.8%.^6^

Symptoms vary depending on the sites of occurrence and include dysmenorrhea, menstrual pain, menstrual irregularities, pelvic pain that is not associated with the menstrual cycle, dyspareunia, defecation pain, and infertility.^1^^,^^7^ Patients sometimes present with a painful mass, premenstrual tenderness, and swelling as the symptoms of endometriosis in the inguinal region.^7^ However, some patients do not exhibit any symptoms such as dysmenorrhea, pelvic pain, and dyspareunia.^4^ Because of variations in the presenting symptoms, heterotopic endometriosis is sometimes misdiagnosed as incarcerated inguinal hernia, lymphadenitis, and hydrocele in the inguinal canal.^4^ Patients with heterotopic endometriosis often have a history of cesarean delivery or surgery for hernias. It is generally thought that surgical chance around the site is one of etiological causes of endometriosis.^4^^,^^7^

MRI is useful for diagnosing heterotopic endometriosis.^7^ It has the ability to identify the presence of iron in hemosiderin deposits contained in the endometrioma and results in a more accurate diagnosis.^7^ However, MRI findings for subcutaneous heterotopic endometriosis are inconsistent and thus diagnostic criteria have not yet been established.^4^^,^^8^ In our case, MRI showed low signal intensity on T1- and T2-weighted images. Ultrasonography, radiological studies, and computed tomography are not useful for diagnosing subcutaneous heterotopic endometriosis.^4^ Furthermore, some cases similar to ours have atypical MRI findings.^4^ The final diagnosis of heterotopic endometriosis is only reached on the basis of a histopathological examination from biopsy or excision at surgery, which demonstrates the histological presence of endometriosis.^4^

Two therapeutic strategies are employed for heterotopic endometriosis: surgery and hormonal therapy. Complete surgical excision is currently the primary treatment to prevent recurrence.^8^

Differential diagnoses of heterotopic endometriosis are incarcerated hernia, femoral hernia, lymphadenopathy, suture granuloma, neuroma, abscess, lymphedema, primary or metastatic cancer, lymphoma, lipoma, hematoma, sarcoma, and subcutaneous cysts.^4^ Although the excision of subcutaneous tumors is a common procedure in plastic and reconstructive surgery fields, difficulties are associated with selecting the correct tumor margin. Therefore, surgeons need to observe the margin carefully and delicately palpate the tumor around the margin. We need to consider the possibility of heterotopic endometriosis when a fertile woman has a painful subcutaneous mass.

## SUMMARY

We herein present a case of heterotopic endometriosis in the inguinal region, which is a rare site of occurrence. Heterotopic endometriosis in the inguinal region needs to be considered as a differential diagnosis when a fertile woman has a painful subcutaneous mass in that site.
